# Discussing spiritual health in primary care and the HOPE tool - a survey of social prescribers

**DOI:** 10.1186/s12875-025-03101-8

**Published:** 2025-12-16

**Authors:** Ishbel Orla Whitehead, Philip Mordue, Mark Adley, Amy O’Donnell, Barbara Hanratty

**Affiliations:** Population Health Sciences Institute, Faculty of Medical Sciences, Campus for Ageing and Vitality, Newcastle upon Tyne, UK

**Keywords:** Spiritual health, Social prescribing, Spirituality, Primary care, Consultation tools

## Abstract

**Background:**

Social prescribing aims to provide holistic care to patients and reduce health inequalities by meeting needs that expand beyond the biomedical model, as detailed in the National Health Service (NHS)’s Long Term Plan. While holistic care includes spiritual health, understanding of social prescribers’ attitudes towards discussing spiritual health with their patients is limited. This study aimed to (1) explore social prescribers’ views on discussing spiritual health with patients; and (2) identify key barriers and facilitators to incorporating spiritual health within their practice, including the potential for using a structured tool (the HOPE tool) to support discussions.

**Method:**

We developed, piloted and administered a bespoke online survey for social prescribers working within primary care in the UK over two months, 1st August to 30th September 2024. This was based on our previous work with UK family doctors (general practitioners or GPs) on the topic of spiritual health and use of spiritual history-taking tools. The acceptability of the HOPE tool was investigated using patient vignettes drawn from clinical practice.

**Results:**

One hundred seventy-one social prescribers responded. One hundred and twenty (70%) reported that they felt comfortable asking patients about their spiritual health. A similar proportion (71%) felt that the HOPE tool was beneficial. Those who were uncomfortable with the topic did not appear to have their comfort affected by learning about the HOPE tool. Attitudes towards spiritual health fell into categories of embracing, pragmatic, guarded and rejecting. Barriers and facilitators to discussion of spiritual health included: a perceived need for patient cues and training; fear of causing offence or upset; and pre-existing practitioner attitudes towards and/or interest in the topic.

**Conclusions:**

Social prescribers are positive about discussing spiritual health with patients and feel comfortable with the topic. The HOPE tool may be helpful where social prescribers are already happy to discuss spiritual health, but other approaches will be needed for those who are reluctant to broach the topic. If social prescribers are more comfortable with the topic of spiritual health than GPs, they may hold the answer to an area of unmet need. Training needs are highlighted for further research.

**Supplementary Information:**

The online version contains supplementary material available at 10.1186/s12875-025-03101-8.

**Table:** 

What is “spiritual health”? Spiritual health is a broad concept, as diverse as people themselves. As part of this survey, spiritual health was not defined and left to the participant to define themselves. The authors use a definition of spiritual health developed by UK General Practitioners: self-actualisation and meaning; transcendence, connectivity and relationships beyond the self; and expressions of spirituality.	

## Introduction

Social prescribing has been developed in UK primary care for patients who need ‘social’ interventions. Social prescribing is currently a major policy focus, with the National Health Service (NHS) committed to embedding social prescribing in primary care as part of its 10-Year Long Term Plan [1]. In social prescribing, a local link worker provides a referral point for General Practitioners (GPs, or family doctors), or other trusted individuals in clinical or community settings [2], to redirect patients who might benefit from non-medical interventions [2]. Social prescribing seeks to improve the health and wellbeing of patients who, for example, have one or more long term health conditions, complex social needs, or need support with low level mental health issues [1, 3].Social prescribers (also called link workers, care coordinators, or other titles) are embedded in their communities and have broad awareness of what is available from community and third sector organisations. They act as a ‘bridge’ between healthcare and community services. Social prescribing aims to facilitate the development of personalised, relational care to meet patients’ holistic needs [4]. It is also a way for GPs to access services that add meaning, purpose and connections to a patient’s life, to improve their holistic health [5]. Meaning, purpose and connections can be considered part of spiritual health [6]. 

Spiritual health is an important part of health and wellbeing, which is defined as not solely being the absence of disease [7]. Existing evidence suggests that spirituality and religiosity can positively impact patients’ health and social care outcomes in multiple ways [8–12]. Conversely, having unmet spiritual needs can be detrimental to health [13–18]. Salutogenesis is a key term used in Social Prescribing literature in the NHS to refer to the ‘creation of health’ [19, 20], and includes the spiritual in its definition [21]. Community faith based organisations could be an overlooked and underutilised health asset [22]. However current social prescribing initiatives do not include any references to spiritual care providers or faith communities [5, 20, 23]. Previous research suggests that GPs in the UK may benefit from training and a concise tool to help overcome the identified barriers to discussing spiritual health [6, 24, 25]. The HOPE (*h*ope; *o*rganised religion; *p*ersonal spiritual practices; *e*ffects on care; see Table 1) [26] tool meets these requirements, as it provides both a clear structure for novice or uncomfortable staff, as well as a flexible and open approach for more experienced practitioners. The initial question is an open, non-religious one, that asks about sources of hope in difficult times.(5) The tool is designed to be used flexibly(5) providing a useful addition to consultation skills rather than a box-ticking exercise [27]. While international studies into the utility of the HOPE tool are ongoing, nothing is known about whether it could be useful within social prescribing [27]. 

**Table 1:** The HOPE tool [26]

There are a few structures or tools suggested to help GPs ask patients about their spiritual health. This survey is about the HOPE tool, developed in the USA, to aid family physicians in taking a spiritual history. The tool provides a series of prompts, and acts as a mnemonic. HOPE stand for:	
**H**	**Hope**- asking patients what gives them hope/sustains them
**O**	**Organised religion**- discussing whether patients interact with any form of organised religion
**P**	**Personal spiritual practice**-
**E**	**Effects on care**- anything the patient needs you to know about how their spirituality impacts on their care, for example at the end of life, or refusal of certain treatments.
**H** ope We have been discussing your support systems. I was wondering what is there in your life that gives you internal support? What are your sources of hope, strength, comfort and peace? What do you hold on to during difficult times? What sustains you and keeps you going? For some people, their religious or spiritual beliefs act as a source of comfort and strength in dealing with life’s ups and downs; is this true for you?	**O** rganised religion Do you consider yourself part of an organized religion? How important is this to you? What aspects of your religion are helpful and not so helpful to you? Are you part of a religious or spiritual community? Does it help you? How?
**P**ersonal spirituality and **p**ractices Do you have personal spiritual beliefs that are independent of organized religion? What are they? Do you believe in God? What kind of relationship do you have with God? What aspects of your spirituality or spiritual practices do you find most helpful to you personally?	**E**ffects on medical care and **e**nd of life issues Has being sick (or your current situation) affected your ability to do the things that usually help you spiritually? (Or affected your relationship with God? ) As a doctor, is there anything that I can do to help you access the resources that usually help you? Are you worried about any conflicts between your beliefs and your medical situation/care/decisions? Would it be helpful for you to speak to a clinical chaplain/community spiritual leader? Are there any specific practices or restrictions I should know about in providing your medical care? (e.g., dietary restrictions, use of blood products) *If the patient is dying*: How do your beliefs affect the kind of medical care you would like me to provide over the next few days/weeks/months?

The aim of this study was to investigate how comfortable social prescribers feel discussing spiritual health with their patients, what the barriers and facilitators could be to the discussion of spiritual health, and to assess the potential benefit of a structured tool (HOPE) to overcome barriers to the discussion of spiritual health within the consultation.

## Method

A mixed methods online survey for social prescribers working in primary care was developed based on our previous survey with GPs [24], piloted with social prescribers, and then refined based on feedback. The survey was distributed to UK National Health Service (NHS) and relevant research organisations using JISC online surveys [28]. Clinical research networks, primary care networks, and social prescribing networks were asked to publicise the survey in newsletters. We also contacted professional online groups and forwarded the link directly to practice managers and GP practices.

Consent was sought online, in writing, prior to the start of the online survey. Questions collected demographic data from the participants, including gender, ethnicity and belief, and occupational characteristics. Participants were asked to use a five-part Likert scale to rate statements about their comfort with asking patients about their spiritual health, including in the context of mental health and end of life care. Participants were asked which, if any, spiritual history-taking tools they were aware of and which they used. The HOPE tool was presented (see Table 1), and participants were asked whether they would feel comfortable using this tool asking patients the questions or comfortable being asked as a patient themselves. Participants were asked for their comfort ratings and comments after each vignette.

Four vignettes (Table 2) were developed from a range of real clinical cases, to reflect the socio-cultural diversity of the United Kingdom (UK), as well as cover scenarios where all main parts of the HOPE tool could be useful.

**Table 2:** Patient vignettes

Patient name	Age	Ethnicity	Religious or similar background	Social issue	Intended spiritual component to consultation
Fatima	32	Arabic name, but used widely	Muslim	Post-natal depression, adjustment and change to identity as a new mum	Isolation, spiritual crisis, mental illness (H, O, P, maybe E)
Olive	72	Unspecified, based on a patient from Europe	Likely Anglican or other mainstream Christian	Loneliness/frailty	Isolation, mild mental illness symptoms, possible functional symptoms.(H, O, P)
Michael	45	Likely British	Jehovah’s Witness	Financial stress, relationship issues, rejection from religious community	Isolation, rejection from organised religion (H, O, maybe P)
David	24	Likely British, ethnic background left open to the reader	Vegan/humanist	Acne, depression	Mental illness, compliance with meds (E)

The participants were asked to use a four-part Likert scale to rate the statements in Table 3 relating to each vignette and provide additional free text comments.

**Table 3:** Statements for rating vignette

1. I would feel comfortable asking this patient about their spiritual health.
2. I think the HOPE tool would be useful with this patient.
3. I would feel comfortable using the HOPE tool with this patient.

### Data analysis

#### Quantitative

Data were extracted from Jisc Online Surveys [28], and analysed using the Stata MP 18.0 package [29]. Logistic regression models were constructed, and McNemar chi squared measure of association calculated if appropriate. Data were aggregated where small numbers required it for statistical analysis, and to preserve participant anonymity.

#### Qualitative

Qualitative data on barriers, facilitators, and use of the HOPE tool in discussion of spiritual health were analysed using a deductive thematic analysis, based upon a priori themes from the literature. A four-step process was used: (13) immersion in the data; stratifying to identify themes by comparing and contrasting similar codes; review of categories; and finally drawing these together to identify the central themes. Two researchers (OW, PM) analysed the data independently and then discussed the findings to reach consensus. Outlying cases were examined to identify insights from those most and least comfortable with the topic.

#### Ethics

Research ethics approval was obtained from Newcastle University on 25th April 2024 and HRA approval was obtained on 31st July 2024, IRAS number 343,749.

### Public and patient involvement (PPI)

PPI work before this project involved members of VOICE, a network of public, patients and carers (https://www.voice-global.org/about/). PPI participants had expressed mixed views about both the topic and the HOPE tool. Some felt HOPE was a respectful and innocuous way to structure a discussion on the topic; all participants asserted that holistic, humanitarian care was essential.

## Results

### Quantitative results

One hundred and seventy-one social prescribers working in primary care responded. The majority were female (85%), based in England (98%) and white (88%) (see Table 4). The majority (112, 65%) described themselves as social prescribers, link workers, or variations of those terms. Other respondents were working as care coordinators (19, 11%), coaches (6, 4%) and in roles using other titles such as community connector or navigator.

**Table 4:** Characteristics of participants

Characteristic (*n* = 171)	Number of Participants	Percentage
Total	171	
Gender		
Female	146	85%
Male	22	13%
Not disclosed or self described	3	2%
Ethnicity		
White background	151	88%
Asian background	10	6%
Black background	4	2%
Mixed or multiple backgrounds	5	3%
Belief related group		
Christian	76	44%
No religion	70	41%
Muslim	9	5%
Humanist	4	2%
Spiritual, not religious	4	2%
Other	2	1%
Prefer not to say	5	3%
Area of UK		
South West of England	39	23%
East Midlands	25	15%
North East England	21	12%
South East England	21	12%
Yorkshire and the Humber	18	11%
East of England	18	11%
London	15	9%
North West England	8	5%
Other areas of England	2	1%
Scotland, Northern Ireland or Wales	4	2%

Most participants suggested they felt comfortable discussing spiritual health with patients in general (119 participants, 70%); 96 participants (56%) felt comfortable discussing spiritual health with people with mental illness; and 96 (56%) were comfortable discussing spiritual health with patients at the end of life. A similar number of social prescribers reported feeling comfortable discussing spiritual health at end of life, and with people with mental illness, however the difference in comfort overall, and comfort with mental health was statistically significant. (χ^2^ = 10, *p* < 0.05). There was no statistically significant difference in comfort with discussing the topic between participants who described themselves as spiritual or not (χ^2^ = 2.90 *p* = 0.09) and those who described themselves as religious or not (χ^2^ = 1.13 *p* = 0.29) (Table 5).

**Table 5:** Baseline comfort with the topic of spiritual health

	Strongly agree	Agree	No opinion	Disagree	Strongly Disagree
I feel comfortable asking patients or clients about their spiritual health.	25 (15%)	94 (55%)	18 (11%)	29 (17%)	5 (3%)
I feel comfortable asking patients or clients about their spiritual health when they are reaching the end of their lives.	27 (16%)	70 (41%)	34 (20%)	31 (18%)	7 (4%)
I feel comfortable asking patients or clients about their spiritual health when they have poor mental health.	22 (13%)	75 (45%)	26 (15%)	37 (22%)	8 (8%)

For all four vignettes, most participants were comfortable discussing spiritual health. Participants felt more comfortable discussing spiritual health than baseline comfort with the topic in the Fatima, Olive and David vignettes. (agreement with “I feel comfortable asking patients or clients about their spiritual health” (*p* < 0.05), shown in Table 6. Participants were less comfortable discussing spiritual health with David and Michael than they were with Olive and Fatima, the difference in comfort with the topic is shown in Table 7.

**Table 6:** Comfort with discussing spiritual health in the patient vignettes, and effect of the HOPE tool

Patient					
*I would feel comfortable asking this patient about their spiritual health*		Fatima	Olive	Michael	David
Agree or strongly agree		151 (89%)	150 (88%)	133 (78%)	118 (69%)
Disagree or strongly disagree		19 (11%)	21 (12%)	38 (22%)	53 (31%)
Number of participants *uncomfortable* discussing spiritual health who *would* be comfortable using the HOPE tool		10 (6%)	8 (5%)	12 (7%)	5 (3%)
Change in agreement with baseline comfort with the topic and comfort with this vignette (McNemar χ^2^)		Relative difference- 0.13 (χ^2^ = 12.46, *p* < 0.05)	Relative difference- 0.11 (χ^2^ = 7.26, *p* < 0.05)	Relative difference- 0.01 (χ^2^ = 0.03, *p* = 0.85)	Relative difference- -0.11 (χ^2^ = 4.24, *p* < 0.05)
*I think the HOPE tool would be useful with this patient*	Agree	154 (91%)	149 (88%)	131 (77%)	108 (63%)
	Disagree	16 (9%)	21 (12%)	40 (23%)	63 (37%)
*I would feel comfortable using the HOPE tool with this patient*	Agree	143 (84%)	141 (82%)	115 (67%)	98 (58%)
	Disagree	28 (6%)	30 (18%)	56 (33%)	72 (42%)

**Table 7:** Differences in comfort discussing spiritual health by vignette

	Fatima	Olive	Michael
Fatima			
Olive	Relative difference- 0.01 Χ^2^ = 0.25 *p* = 0.6		
Michael	Relative difference- 0.13 Χ^2^ = 12.46 *p* < 0.05	Relative difference- 0.11 Χ^2^ = 8.76 *p* < 0.05	
David	Relative difference- 0.22 Χ^2^ = 26.56 *p* < 0.05	Relative difference- 0.27 Χ^2^ = 22.26 *p* < 0.05	Relative difference- 0.11 Χ^2^ = 5.49 *p* < 0.05

### The HOPE tool

#### Use of history taking tools and comfort with the HOPE tool

The majority (86%) of respondents stated that they never use a tool to support discussion of spiritual health. Most (82%) would be comfortable being asked the questions in the HOPE tool as a patient, and 67% would feel comfortable using the HOPE tool with their patient. Most (52%) had no opinion on whether the HOPE tool would protect them from allegations, and concern over allegations did not appear to feature in the qualitative analysis either. One hundred and thirty three participants (78%) felt that the HOPE tool would provide a useful documentation structure. Respondents who reported discomfort with the topic of spiritual health were also uncomfortable with use of the HOPE tool (McNemar χ^2^ = 0.47 *p* = 0.49).

### Qualitative results and analysis

#### Attitudes towards discussing spiritual health with patients

Participants mentioned the diversity of patients that they work with in terms of identity and culture and reported that they favoured a patient-led approach to their practice, allowing patients to guide the conversation. Attitudes towards the discussion of spiritual health closely mirrored existing evidence [6, 30]: embracing, pragmatic, guarded and rejecting, shown in Table 8.

**Table 8:** Attitudes to spiritual health

Attitude	Description	Examples
Embracing	Participants described asking about spiritual health as part of their routine practice. This was usually where it was part of a form, or their own personal interest.	“I fully advocate for all of the above if I feel the patient will be receptive to it.” “I often refer people for reiki or healing. I have also referred people to yoga and meditation classes for support with mental health.”
Pragmatic	Participants focused on their ‘patient-led’ role, and that if a patient raised the topic they would be happy to explore and support these needs.	“If a patient expresses that they belong to a faith group, I will encourage them to connect with it as part of their support network.” “I am led by the patient, if they have these beliefs and interest, then I would work with them on how to connect to these.”
Guarded	Participants were more cautious than the reactive group, and were less comfortable, even when a patient rose the topic.	“I will only talk about it if my patient brings it up first.” “I would not recommend a specific place of worship but would advise patients to explore the options independently.”
Rejecting	These participants felt that ‘religion and politics’ were off limits, and that spiritual health was not part of their role.	“I feel it is not my place to suggest someone attends a religious organisation as I have no understanding of people’s religious beliefs.” “I would prefer not to have discussions with patients about religion or politics as I’m not educated enough in either topic.”

#### Barriers and facilitators to discussing spiritual health

**Fig. 1:**
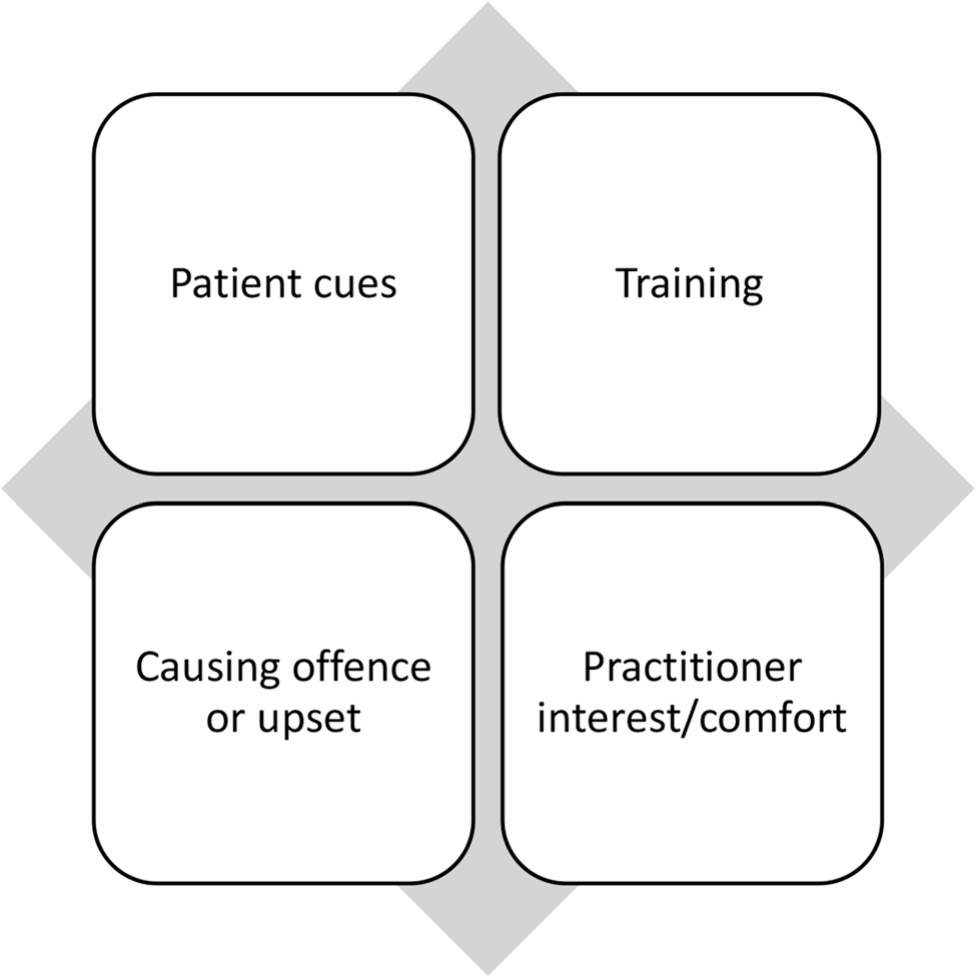
Themes of barriers and facilitators to the discussion of spiritual health for social prescribers

The themes identified of barriers and facilitators to discussion spiritual health were a need for a patient cue; a need for training; a fear of causing offence or disrupting the relationship with the patient; and practitioner interest and/or comfort with the topic of spiritual health (Fig. 1).

#### Patient cues

This was a core issue raised throughout the responses. Participants took pride in their open, patient- led, holistic approach, and unless asking about spiritual health was part of their usual routine, they waited for a patient cue that the topic was important. Cues from patients included both verbal and non-verbal, for example, language used contextually, or iconography in the home.

“Whether they bring it up, which they often don’t—I work to a model of ‘what matters to the patient’.” (Participant 45, female, no religion, spiritual)

“People are generally very open about religious faith and do bring this up… Iconography around the home also prompts discussion.” (Participant 73, female, Christian)

“It depends on what the patient says. By the way they talk, some words would usually tell me if they have a faith.” (Participant 120, female, Christian, spiritual)

“Sometimes, a patient might mention that they haven’t been to the church coffee morning for a while, so I would go back to that statement and start the conversation from there.” (Participant 61, female, no religion, not spiritual)

#### Training

Most participants felt they needed training to improve competence and confidence. Specifically, participants felt that they did not know enough about the diversity of religions and approaches to spiritual health. The HOPE tool could be a useful part of training.

“I would want to feel confident and have training to ensure that we are effective in our understanding of spiritual health.” (Participant 80, female, Christian)

“ I would like some training around using the HOPE tool.” (Participant 25, female, no religion, not spiritual)

In answer to ‘What affects whether you discuss spiritual health?’: “Different views and worrying about offending a patient due to lack of knowledge on their religion.” (Participant 10, female, no religion, spiritual).

Participants did appear to be positive about future learning and training on spiritual health:

“This questionnaire has made me think more about spiritual health, and I will enjoy doing more research.” (Participant 168, female)

#### Practitioner interest/comfort

Respondents who appeared most active in their discussion of spiritual health were those who felt competent and asked about spirituality or religion as part of their usual routine. Personal practitioner comfort with the topic appeared to be both a barrier (if uncomfortable) and a facilitator (if confident and/or interested in the topic) to discussing spiritual health with patients.

“I find spiritual practices such as yoga and meditation easier to explore/suggest to patients, perhaps because it aligns with my beliefs.” (Participant 82, female, Christian, spiritual)

“Personally, I have trauma associated with religion, so I feel uncomfortable in certain religious settings.” (Participant 79, female, Christian, spiritual)

#### Causing offence or upset to patients

Participants described spiritual health as a personal and sensitive topic. Some participants mentioned the effects of religious trauma, especially in minority or vulnerable groups. They were wary of alienating patients, of undermining their working relationship, or crossing professional boundaries.

“Some patients have negative experiences with religion, so discussing spiritual health can be distressing for them.” (Participant 162, female, Humanist, not spiritual)

“I worry that if I raise spirituality, it may encourage some patients to disengage with the service.” (Participant 56, female, no religion, not spiritual)

“I do not want to be accused of exploiting or manipulating people, or taking advantage of anyone.” (Participant 149, female, Muslim, spiritual))

#### The HOPE tool

Participants had mixed views on the use of the HOPE tool. When offering thoughts on training in the topic of spiritual health, the HOPE tool was viewed as a positive option, providing structure and clarity through a holistic framework for discussion. Others felt that the tool may make the topic less taboo. Comments on its limitations included the time taken for its use, a feeling that it was “intense” (participant 155) and whether asking structured in-depth questions would move the conversation away from being fully patient-led.

“If I had the HOPE tool to hand and felt confident with using it, I think that kind of conversation with the patient would be of high benefit” (Participant 15, female, no religion, not spiritual)

Participants suggested that an adaptable and flexible approach to the tool could be beneficial. The H (hope) and P (personal spiritual practice) parts of the tool were felt to be less invasive and more useful to promote patient centred discussion. Some suggested that the HOPE tool could be used to add dimensions to tools they already use, such as ‘Wheels of Wellbeing’, which appear to be locally decided frameworks and vary as to whether they include aspects of spiritual health [31–33]. 

## Discussion

### Summary of main findings

This large survey examined the views of social prescribers working in primary health on discussing spiritual health with patients. We found that many social prescribers were receptive to discussing the topic of spiritual health, but generally if it was part of their usual routine, if they had a personal interest, or if a patient gave a cue that they had spiritual health needs. This reticence to raise the topic of spiritual health may lead to unmet needs if the patient is waiting for the practitioner to raise the topic [34], therefore an argument can be made for gentle enquiry from practitioners. Participants were broadly comfortable with the idea of discussing spiritual health with patients, whether or not they saw themselves as spiritual people. However, when vignettes were used to test comfort, participants reported being more comfortable with the female than the male vignettes, and were less comfortable discussing spiritual health with the ‘David’ vignette, who was not religious, but had a strong ethical code (veganism). It is unknown whether this was due to gender, or the lack of religious cue, or another reason, such as discordance between participant and the vignette. Participants reported being more comfortable discussing spiritual health overall than in the specific cases of the end of life, or with in mental health, although further research is needed to explore why this would be, given the higher spiritual needs of these populations [35, 36]. 

Some participants reported strong personal feelings about their personal religion and sense of vocation, and embraced the topic, while a small minority rejected the concept of spiritual health. However, most participants described what spiritual health meant to them and felt comfortable with the topic. While the HOPE tool was viewed positively by participants, due to high baseline comfort with the topic, use of the HOPE tool did not increase comfort with the topic.

Improved training in the topic is likely to be necessary to increase comfort with the discussion of spiritual health. While the HOPE tool may provide a useful framework for discussion, it may be too in-depth in its entirety for social prescribing consultations, and use of a tool does not appear to have much effect on comfort in this group of professionals. Those who were uncomfortable with the topic of spiritual health were also uncomfortable with the HOPE tool suggests that a tool alone may not be enough to overcome these participants’ reluctance to discuss this topic with patients. It will be important that any training contains an overview of common religions and other belief-based cultures and practices, and improves confidence and competence and reduces concerns about causing offence.

### Comparison with other work

As far as we are aware, this is the first study in which social prescribers have been asked about spiritual health and how the topic fits into their practice. Social prescribing has been suggested previously as a way for patients to access support for spiritual health needs identified by GPs [24]. As noted above, social prescribers’ attitudes are in line with those of general practitioners in Scotland [30]. However social prescribers appear to feel much more comfortable than GPs with the idea of discussing spiritual health (70% compared with 49%), as well as more comfortable when given patient vignettes.

The attitudes of social prescribers are similar to the embracing, pragmatic, guarded and rejecting attitudes found in previous work with GPs [6, 30]. We found social prescribers pride themselves on being patient-led in their discussions, and fear causing offence to patients, or harm to those with religious trauma.

Social prescribers here, and in previous research, place importance on the interpersonal relationship and the patient-centred approach [37]. It follows that the need for a patient cue to introduce the topic is prominent in this study. This has been identified previously with other primary care workers [24, 26] Whereas GPs were concerned about allegations of professional misconduct when discussing spiritual health [24], social prescribers do not appear to have the same concerns, although they do worry about causing offence to patients. In findings that are similar to those of a previous survey with GPs [24], social prescribers feel that they need more training in the topic, and that some aspects of the HOPE tool could be beneficial [24]. 

### Strengths and limitations

The survey attracted a large response from social prescribers working within primary health. Views expressed were varied and frank regarding where spiritual health fits, or not, in current social prescribing practice. The study was designed and conducted with patient and public participation, which should ensure that it remained patient focussed. However, respondents were a self-selected sample, and strong views may have prompted participation. The respondents were mostly female and white, and while this may reflect the profile of a majority of English social prescribers there are no national data to confirm this. The majority of respondents were from England, limiting the conclusions that could be applied to other nations in the UK. This was despite targeted efforts such as phone calls and additional invites. The response rate may reflect variation in social prescribing capacity in the different nations. s. Non-Christian religions were combined due to small numbers, limiting analysis of the effect of religious affiliation.

### Implications for research and practice

This study shows that there are training needs for social prescribers around the topic of spiritual health. Social prescribing consultations are patient-led, and many social prescribers are either reactive or passive in their approach and would discuss spiritual health needs if the patient raised them. However, a reliance on patients to raise the topic may generate inequities in addressing spiritual health needs.(11) The range of attitudes from social prescribers could lead to inequity of access to support for patients with spiritual health needs. This inequity of access to support could be exacerbated by variation in practitioner interest and competence in the topic. Given the correlations identified between a high level of spiritual health and positive mental health status [38], and the recent calls for spirituality to be considered as a determinant of health [39], further research into training for the primary health care team on the discussion of spiritual health in primary care is warranted.

## Conclusions and recommendations

Social prescribing aims to be an holistic approach to wider health and wellbeing, but current approaches and attitudes of social prescribers may be causing barriers to the discussion of spiritual health and the identification of spiritual health needs in patients.

This study suggests that training for social prescribers may be helpful. This could enable them to address spiritual health as a routine, practitioner led topic and to overcome barriers such as fear of causing offence. A modified or shortened version of the HOPE tool may be useful for this. These data describe similar views to those of GPs, and suggest that the training needs could be team-wide. Further information is needed to identify how social prescribers define spiritual health, and their current practice and relationships with spiritual, religious and faith based community organisations. Our future work will explore this through interviews with social prescribers.

Spiritual health is a key aspect of healthcare that can be neglected and avoided due to practitioners waiting for the patient to raise the topic, therefore this may lead to unidentified health needs. With the widening of access to social prescribing, social prescribers are uniquely placed, by being embedded in the community, to be aware of community faith, and non-faith, based provision that could meet unmet spiritual health needs. We suggest further work in the area of spiritual health to bring together community providers, and healthcare, via social prescribing.

## Supplementary Information

Supplementary Material 1.

## Data Availability

Data are saved on Newcastle University secure servers and may be available in negotiation with the first author. While participants were not consented to allow public sharing of this data, data are available upon reasonable request to the authors. Whitehead, Ishbel (2025). Defining and discussing spiritual health in primary care and the HOPE tool - A survey of social prescribers.

## Abbreviations

GP: General Practitioners
NHS: National Health Service
HOPE: the HOPE tool [26]
PPI: Public and Patient Involvement
