# Supplementary material for: Discussing spiritual health in primary care and the HOPE tool - a survey of social prescribers
Source: BMC Prim Care. 2025 Dec 16;26:399. doi: 10.1186/s12875-025-03101-8 (PMC12709808; doi:10.1186/s12875-025-03101-8)
Supplement: Supplementary file 1 — Supplementary Material 1. [file 12875_2025_3101_MOESM1_ESM.pdf]

Discussing Spiritual Health in Primary Care- social prescribing - Duplicate

1. Do you work in social prescribing in a non-clinical role?
   This includes anyone who works in a role that bridges community networks and provision, and prim
   could have a variety of names.
   For example as a community connector, link worker, care coordinator, social prescriber, or another r
   primary care and the community?
   https://www.england.nhs.uk/personalisedcare/social-prescribing/#:~:text=Social%20prescribing%20is%20a%20key,affect%20their%20health%20and

◯ Yes

◯ No

Information Sheet for those involved in Social prescribing or similar, in UK Primary Care Title of Study: SHARP- Spiritual Health Assessment and Referral in Primary Care 18th July 2024 Version 4

IRAS 343749

Invitation and Brief Summary

You are being invited to take part in a research study. You do not have to take part. Before you decide whether or not you wish to take part it is important that you understand why the research is being done and what it will involve. Please read this information carefully and discuss it with others if you wish. Take time to decide whether or not you wish to take part. If you do decide to take part, you will be asked to complete an online consent form. However, you are free to withdraw at any time, without giving any reason and without any penalty or loss of benefits.

In this research study we will use information from you. We will only use information that we need for the research study. We will let very few people know your name or contact details, and only if they really need it for this study.

Everyone involved in this study will keep your data safe and secure. We will also follow all privacy rules.

At the end of the study we will save some of the data in case we need to check it, and for future research.

We will make sure no-one can work out who you are from the reports we write.

What is the purpose of the research? Spiritual health is an important part of holistic, or whole person, health. Religiosity and spirituality are associated with a longer life, and can beneficial for health, including those with mental illness, HIV and dementia. Attending religious services may also enhance rehabilitation after cardiac illness. Unmet spiritual needs can be detrimental to health, including greater pain and lower wellbeing. If good spiritual health does lead to a longer and/or healthier life, then patients may need us to consider that with them. To aid this, various tools have been created, and the HOPE tool may be helpful. This research seeks to get the views of social prescribing link workers, care coordinators, care navigators and their teams regarding discussing spiritual health, and the use of the HOPE tool. The HOPE tool has been chosen as it is felt it is memorable, short, flexible and applicable to a diverse population.

Who is funding this research? This research is funded by The John Templeton Foundation, USA, whose ethos of scientific inquiry to promote interdisciplinary research to promote human flourishing

mirrors that of the research team. The foundation has no influence over the findings of the study.
What does taking part involve? This research involves an online questionnaire. The questions ask for some basic information about you, and the population you serve. Further questions will ask you about your comfort and experiences of taking a spiritual history with patients or clients. What information will be collected and who will have access to the information collected? Anonymised demographic information will be collected, including your: ethnicity, gender, religion or belief, type of population served, how long you have worked in social prescribing, or similar, your job title, or role. This information is needed to see whether these factors relate to how comfortable those involved in social prescribing are discussing spiritual health. You will then be given an explanation of a history taking tool, and asked to rate how comfortable you feel using these tools in some scenarios given. There will be a chance for you to give comments. Religion and spirituality can be a sensitive issue, and personal questions can be left unanswered should you wish. The data will be handled by the researcher, and will be stored securely and electronically. Any personal or contact details will be stored separately to your responses. If you wish, we will use your email address to provide further information about the research study. Individuals at Newcastle University may look at your anonomised research data to check the accuracy of the research study. If you agree to take part in the research study, your anonomised data will become part of a dataset which can be accessed by other users running other research studies at Newcastle University and in other organisations. These organisations may be universities, or NHS organisations. Your information will only be used by organisations and researchers to conduct research. The information will only be used for the purpose of research, and cannot be used to contact you. It will not be used to make decisions about future services available to you. During the survey, you can withdraw at any time, and no data will be saved. After completion, as the survey is anonymous, it will not be possible with withdraw your data.

Why have I been invited to take part? You have been invited because we believe you are involved in social prescribing or a similar role, for example, social prescribing link worker, a care coordinator, navigator, other person involved in social prescribing, or part of a social prescribing team, working alongside general practice or primary care, in the UK.
What are the possible benefits of taking part? You will be contributing to a study which will help enhance our understanding of discussing spiritual health with our patients. If you take part, you will read a description of a tool to help with discussing spiritual health in practice. This may help guide your continuing professional development.
What are the possible disadvantages and risks of taking part? No known risks. It will take a few minutes of your time.
Who is the sponsor and data controller for this research? Newcastle University is the sponsor for this study based in the United Kingdom. Newcastle University will be using information from you in order to undertake this study and will act as the data controller for this study. This means that Newcastle University is responsible for looking after your information and using it properly. The lawful basis for carrying out this study under GDPR is Task in the Public Interest, (Article 6,1e) as research is cited as part of the University's duties. Your rights to access, change or move your information are limited, as Newcastle University need to manage your information in specific ways in order for the research to be reliable and accurate. If you withdraw from the study, Newcastle University will keep the information about you that has already been obtained.
How will we use information about you?
We will need to use information from you for this research project.

This information will not include any identifiable information. People will use this information to do the research or to check your records to make sure that the research is being done properly.

We will keep all information about you safe and secure.

Once we have finished the study, we will keep some of the data so we can check the results. We will write our reports in a way that no-one can work out that you took part in the study.

What are your choices about how your information is used?
You can stop being part of the study at any time, without giving a reason, but we will keep information about you that we already have.
We need to manage your records in specific ways for the research to be reliable. This means that we won't be able to let you see or change the data we hold about you.
You can find out more about how Newcastle University uses your information at https://www.ncl.ac.uk/data.protection/policy.htm and/or by contacting their Data Protection Officer by emailing rec-man@ncl.ac.uk
Has this study received ethical approval? This study has received ethical approval from Newcastle University Committee on 25th April 2024.
Who should I contact for further information relating to the research? Orla Whitehead orlawhitehead@newcastle.ac.uk
Who should I contact in order to file a complaint? Barbara Hanratty, Newcastle University, Barbara.Hanratty@newcastle.ac.uk If you wish to raise a complaint on how your personal data is handled, you can contact the Data Protection Officer, Maureen Wilkinson, who will investigate the matter: rec-man@ncl.ac.uk If you are not satisfied with their response you can complain to the Information Commissioner's Office (ICO): https://ico.org.uk/

Os na allwch gwblhau'r arolwg hwn yn Saesneg, anfonwch e-bost orla.whitehead@newcastle.ac.uk

Consent Form

1. I confirm that I have read the preceding information sheet dated 18th July 2024 for the above study, I have had the opportunity to consider the information, ask questions and I have had any questions answered satisfactorily.
2. I understand that my participation is voluntary and that I am free to withdraw at any time without giving any reason. I understand that if I decide to withdraw from the questionnaire, any data that I have provided up to that point will be excluded. However, if the questionnaire has been submitted, the data will be included.
3. I consent to the processing of my personal information such for the purposes of this research study, as described in the information sheet preceding this consent form.
4. I consent to my anonymised research data being stored and used by others for future research.
5. I understand that my research data may be published as a report.
6. If I give my email address, I consent to the retention of my email address for 26 weeks, for the purpose of being re-contacted.
7. I understand that my anonomised research data may be looked at by individuals from Newcastle University where it is relevant to my taking part in this research.
8. I agree to take part in this research project.

I agree with points 1-8 above, and consent to be included in the study:
Yes to all

2. I agree with points 1-8 above, and consent to be included in the study: *

○ Yes to all

HOPE tool

There are a few structures or tools suggested to help GPs ask patients about their spiritual health. This survey is about the HOPE tool, developed in the USA, to aid family physicians in taking a spiritual history. The tool provides a series of prompts, and acts as a mnemonic.

HOPE stands for:

H - Hope- asking patients what gives them hope/sustains them

O- Organised religion- discussing whether patients interact with any form of organised religion

P- Personal spiritual practice

E- Effects on care- anything the patient needs you to know about how their spirituality impacts on their care, for example at the end of life, or refusal of certain treatments.

Here are some examples of ways questions can be asked:

Hope: We have been discussing your support systems. I was wondering what is there in your life that gives you internal support? What are your sources of hope, strength, comfort and peace? What do you hold on to during difficult times? What sustains you and keeps you going? For some people, their religious or spiritual beliefs act as a source of comfort and strength in dealing with life's ups and downs; is this true for you?

Organised religion: Do you consider yourself part of an organized religion? How important is this to you? What aspects of your religion are helpful and not so helpful to you? Are you part of a religious or spiritual community? Does it help you? How?

Personal spirituality and practices: Do you have personal spiritual beliefs that are independent of organized religion? What are they? Do you believe in God? What kind of relationship do you have with God? What aspects of your spirituality or spiritual practices do you find most helpful to you personally?

Effects on medical care and end of life issues: Has being sick (or your current situation) affected your ability to do the things that usually help you spiritually? (Or affected your relationship with God?) As a doctor, is there anything that I can do to help you access the resources that usually help you? Are you worried about any conflicts between your beliefs and your medical situation/care/decisions? Would it be helpful for you to speak to a clinical chaplain/community spiritual leader? Are there any specific practices or restrictions I should know about in providing your medical care? (e.g., dietary restrictions, use of blood products) If the patient is dying: How do your beliefs affect the kind of medical care you would like me to provide over the next few days/weeks/months?

The whole tool typically takes 5-6 minutes in a consultation.

3. Please rate the following statements *

I feel comfortable asking myself the questions in the HOPE tool

- Strongly agree
- Agree
- No opinion
- Disagree
- Strongly disagree

I would be comfortable as patient or client being asked the HOPE tool questions by a professional

- Strongly agree
- Agree
- No opinion
- Disagree
- Strongly disagree

I would be comfortable using the HOPE tool with my patients or clients

- Strongly agree
- Agree
- No opinion
- Disagree
- Strongly disagree

I would find HOPE a useful structure for documenting discussions of spiritual health

- Strongly agree
- Agree
- No opinion
- Disagree
- Strongly disagree

I feel using a tool like HOPE would be beneficial

- Strongly agree
- Agree
- No opinion
- Disagree
- Strongly disagree

I feel using a tool like HOPE would protect me from allegations

- ◯ Strongly agree
- ◯ Agree
- ◯ No opinion
- ◯ Disagree
- ◯ Strongly disagree

A reminder of the HOPE tool is at the bottom of every page.

Initial Questions

4. What is your role title? For example 'link worker', 'care coordinator' etc? *

5. Are you:

- Female
- Male
- Prefer to self describe
- Prefer not to say

6. Please self describe if you wish:

7. What is your ethnic group?

- White English / Welsh / Scottish / Northern Irish / British
- White Irish
- White Gypsy or Irish Traveller
- Any other White background, please describe
- White and Black Caribbean
- White and Black African
- White and Asian
- Any other Mixed / Multiple ethnic background, please describe
- Indian
- Pakistani
- Bangladeshi
- Chinese
- Any other Asian background, please describe
- Black African

- ◯ Black Caribbean
- ◯ Any other Black / African / Caribbean background, please describe
- ◯ Arab
- ◯ Any other ethnic group, please describe

8. If you selected Other, please specify:

9. Would you describe yourself as being part of one of these belief related groups?

- ◯ No religion
- ◯ Christian
- ◯ Muslim
- ◯ Jewish
- ◯ Sikh
- ◯ Buddhist
- ◯ Other (please write)
- ◯ Humanist
- ◯ Prefer not to say

10. If you selected Other, please specify:

11. Which area do you currently work in? *

- ◯ Scotland
- ◯ North East England
- ◯ North West England
- ◯ Yorkshire and the Humber
- ◯ East Midlands
- ◯ West Midlands
- ◯ East of England

- ◯ South East England
- ◯ London
- ◯ South West of England
- ◯ Northern Ireland
- ◯ Wales
- ◯ Other

12. If you selected Other, please specify:

13. How many years have you worked in social prescribing? *

- ◯ 0-1
- ◯ 2-3
- ◯ 3-4
- ◯ 5-6
- ◯ 7+

14. Where do you work? *

- ☐ Rural area

☐ Urban area

15. Please rate the following about yourself: *

I am a spiritual person

- ◯ Strongly agree
- ◯ Agree
- ◯ No opinion
- ◯ Disagree
- ◯ Strongly disagree

I am a religious person

- ◯ Strongly agree
- ◯ Agree
- ◯ No opinion
- ◯ Disagree
- ◯ Strongly disagree

I am spiritually healthy

- ◯ Strongly agree
- ◯ Agree
- ◯ No opinion
- ◯ Disagree
- ◯ Strongly disagree

I feel comfortable asking patients or clients about their spiritual health

- ◯ Strongly agree
- ◯ Agree
- ◯ No opinion
- ◯ Disagree
- ◯ Strongly disagree

I feel comfortable asking patients or clients about their spiritual health, when they have poor mental health.

- ◯ Strongly agree
- ◯ Agree
- ◯ No opinion
- ◯ Disagree
- ◯ Strongly disagree

I feel comfortable asking patients or clients about their spiritual health when they are reaching the end of their lives.

- ◯ Strongly agree
- ◯ Agree
- ◯ No opinion
- ◯ Disagree
- ◯ Strongly disagree

16. What does the term 'Spiritual Health' mean to you?

17. Do you ever discuss spiritual health with a patient or client?

- ☐ Always
- ☐ Often
- ☐ Sometimes
- ☐ Rarely
- ☐ Never

18. What affects whether you discuss spiritual health with your patients/clients?

19. Do you ever suggest that patients or clients use community spiritual or religious resources? For example, suggest they could see a religious or spiritual leader or worker, attend religious or spiritual practice (church, temple, meditation, yoga, nature group with a spiritual element).

- ☐ Always
- ☐ Often
- ☐ Sometimes
- ☐ Rarely

☐ Never

20. Why would you use, or not use, community religious and/or spiritual resource providers?   These include places of worship, religious organisations, humanist organisations, religious or spiritually based practice such as yoga or meditation or sound baths, nature activities with a spiritual element, anything that mentions spirit, soul, spirituality or religion.

Why would you use, or not use, community religious and/or spiritual resource providers?

These include places of worship, religious organisations, humanist organisations, religious or spiritually based practice such as yoga or meditation or sound baths, nature activities with a spiritual element, anything that mentions spirit, soul, a deity, connections to something a bigger, a higher power, spirituality or religion.

21. If you have identified a spiritual health need, are you confident that you know how best to discuss this with a patient?

☐ Always
☐ Often
☐ Sometimes
☐ Rarely
☐ Never

22. Please select any spiritual history taking tools you have heard of: *

☐ BELIEF
☐ SPIRITual History
☐ FICA
☐ HOPE
☐ FAITH
☐ None of the above

23. How often do you use a spiritual history taking tool.

☐ Always
☐ Very Frequently

- [ ] Occasionally
- [ ] Rarely
- [ ] Very Rarely
- [ ] Never

24. If you do use a spirirual history taking tool, which one(s) do you use?

- [ ] FICA
- [ ] BELIEF
- [ ] SPIRITual history
- [ ] HOPE
- [ ] FAITH
- [ ] LOADS SHARED

Vignette One

Fatima, a 32 year old first time mum, saw her GP for her post-natal check. She had a difficult delivery, but she and the baby are now physically well. She is struggling to breastfeed, and discloses that she is very anxious, irritable and tearful. The GP thought that the social prescribing team could help her, and put her in touch with you.
Fatima explains that before starting maternity leave, she worked for an Islamic company, and felt well supported. Since having her baby, she is tearful, and feels isolated, struggling with her prayers.

25. Please rate the following statements: *

I would feel comfortable asking this patient about their spiritual health

- ◯ Strongly agree
- ◯ Agree
- ◯ Disagree
- ◯ Strongly disagree

I think the HOPE tool would be useful with this patient

- ◯ Strongly agree
- ◯ Agree
- ◯ Disagree
- ◯ Strongly disagree

I would feel comfortable using the HOPE tool with this patient

- ◯ Strongly agree
- ◯ Agree
- ◯ Disagree
- ◯ Strongly disagree

26. Any further comments?

HOPE stands for: H - Hope- asking patients what gives them hope/sustains them O- Organised religion- discussing whether patients interact with any form of organised religion P- Personal spiritual practice E- Effects on care- anything the patient needs you to know about how their spirituality impacts on their care, for example at the end of life, or refusal of certain treatments.

Vignette Two

Olive is a 72 year old woman with chronic obstructive pulmonary disease (COPD.) She has been referred to you as the GP has noticed she is asking for home visits, and the GP feels Olive may have some social reasons for this. You visit her at home. She is very happy to see you, and tries to keep you chatting for as long as possible. She mentions that she is very lonely since her husband died, and her children live far away.
Olive tells you that she grew up abroad, as her parents were missionaries. She then worked for the civil service abroad, including in Russia. She and her husband returned to the UK when they had children, and her husband sang in the local choir.

27. Please rate the following statements: *

I would feel comfortable asking this patient about their spiritual health

- Strongly agree
- Agree
- Disagree
- Strongly disagree

I think the HOPE tool would be useful with this patient

- Strongly agree
- Agree
- Disagree
- Strongly disagree

I would feel comfortable using the HOPE tool with this patient

- Strongly agree
- Agree
- Disagree
- Strongly disagree

28. Any further comments?

HOPE stands for: H - Hope- asking patients what gives them hope/sustains them O- Organised religion- discussing whether patients interact with any form of organised religion P- Personal spiritual

practice E- Effects on care- anything the patient needs you to know about how their spirituality impacts on their care, for example at the end of life, or refusal of certain treatments.

Vignette Three

Michael, a 45 year old man, has been referred to you for some help with financial stress, after he saw his GP with erectile dysfunction.
You notice that Michael has 'declines all blood products' on his medical notes, and ask if he is a Jehovah's Witness. He explains he used to be, until he was asked to leave the fellowship for leaving his wife, and starting a new relationship 2 months ago.

29. Please rate the following statements: *

I would feel comfortable asking this patient about their spiritual health

- ◯ Strongly agree
- ◯ Agree
- ◯ Disagree
- ◯ Strongly disagree

I think the HOPE tool would be useful with this patient

- ◯ Strongly agree
- ◯ Agree
- ◯ Disagree
- ◯ Strongly disagree

I would feel comfortable using the HOPE tool with this patient

- ◯ Strongly agree
- ◯ Agree
- ◯ Disagree
- ◯ Strongly disagree

30. Any further comments?

HOPE stands for: H - Hope- asking patients what gives them hope/sustains them O- Organised religion- discussing whether patients interact with any form of organised religion P- Personal spiritual practice E- Effects on care- anything the patient needs you to know about how their spirituality impacts on their care, for example at the end of life, or refusal of certain treatments.

Vignette Four

David is a 24 year old man saw his GP with moderate to severe acne. He was referred to you because he keeps coming back to the GP, despite the GP prescribing him medications. The GP wonders if there is 'more' going on. David tells you his acne is affecting his self-esteem, and he feels low, even suicidal at times.
David explains he has been prescribed lymecycline and fluoxetine capsules, but has taken neither, as he is vegan. He mentions he is interested in conservation.

31. Please rate the following statements: *

I would feel comfortable asking this patient about their spiritual health

- ◯ Strongly agree
- ◯ Agree
- ◯ Disagree
- ◯ Strongly disagree

I think the HOPE tool would be useful with this patient

- ◯ Strongly agree
- ◯ Agree
- ◯ Disagree
- ◯ Strongly disagree

I would feel comfortable using the HOPE tool with this patient

- ◯ Strongly agree
- ◯ Agree
- ◯ Disagree
- ◯ Strongly disagree

32. Any further comments?

HOPE stands for: H - Hope- asking patients what gives them hope/sustains them O- Organised religion- discussing whether patients interact with any form of organised religion P- Personal spiritual practice E- Effects on care- anything the patient needs you to know about how their spirituality impacts on their care, for example at the end of life, or refusal of certain treatments.

If you would be interested in further research on this topic, for example interviews, or further information, please email orla.whitehead@newcastle.ac.uk
